# Test characteristics of the SD FK80 *Plasmodium falciparum*/*Plasmodium vivax *malaria rapid diagnostic test in a non-endemic setting

**DOI:** 10.1186/1475-2875-8-262

**Published:** 2009-11-22

**Authors:** Philippe Gillet, David PJ van Dijk, Emmanuel Bottieau, Lieselotte Cnops, Marjan Van Esbroeck, Jan Jacobs

**Affiliations:** 1 Department of Clinical Medicine, Institute of Tropical Medicine (ITM), Nationalestraat 155, B 2000 Antwerp, Belgium; 2 Faculty of Health, Medicine and Life Sciences (FHML), Maastricht, The Netherlands

## Abstract

**Background:**

The SD FK80 P.f/P.v Malaria Antigen Rapid Test (Standard Diagnostics, Korea) (FK80) is a three-band malaria rapid diagnostic test detecting *Plasmodium falciparum *histidine-rich protein-2 (HRP-2) and *Plasmodium vivax*-specific lactate dehydrogenase (Pv-pLDH). The present study assessed its performance in a non-endemic setting.

**Methods:**

Stored blood samples (n = 416) from international travellers suspected of malaria were used, with microscopy corrected by PCR as the reference method. Samples infected by *Plasmodium falciparum *(n = 178), *Plasmodium vivax *(n = 99), *Plasmodium ovale *(n = 75) and *Plasmodium malariae *(n = 24) were included, as well as 40 malaria negative samples.

**Results:**

Overall sensitivities for the diagnosis of *P. falciparum *and *P. vivax *were 91.6% (95% confidence interval (CI): 86.2% - 95.0%) and 75.8% (65.9% - 83.6%). For *P. falciparum*, sensitivity at parasite densities ≥ 100/μl was 94.6% (88.8% - 97.6%); for *P. vivax*, sensitivity at parasite densities ≥ 500/μl was 86.8% (75.4% - 93.4%). Four *P. falciparum *samples showed a Pv-pLDH line, three of them had parasite densities exceeding 50.000/μl. Two *P. vivax *samples, one *P. ovale *and one *P. malariae *sample showed a HRP-2 line. For the HRP-2 and Pv-pLDH lines, respectively 81.4% (136/167) and 55.8% (43/77) of the true positive results were read as medium or strong line intensities. The FK80 showed good reproducibility and reliability for test results and line intensities (kappa values for both exceeding 0.80).

**Conclusion:**

The FK80 test performed satisfactorily in diagnosing *P. falciparum *and *P. vivax *infections in a non-endemic setting.

## Background

Malaria rapid diagnostic tests (RDTs) are immunochromatographic tests that detect circulating antigens of *Plasmodium *species. They are an adjunct to the microscopic diagnosis of malaria, both in the endemic and in the non-endemic settings [1-5]. RDTs are built on a nitrocellulose platform and are available in dipstick or cassette format. Signals are visible as coloured lines, comprising a control line (which indicates that the test has been performed well) and one or two test lines. The so-called two band tests generate a test line that targets *P. falciparum *by detecting either histidine-rich protein 2 (HRP-2) or *P. falciparum*-specific parasite lactate dehydrogenase (Pf-pLDH). The three band tests include a second target that is common to the four *Plasmodium *species, such as aldolase or pan-specific parasite lactate dehydrogenase (pan-pLDH). Apart from these common formats, there are also two-band tests detecting *Plasmodium vivax*-specific pLDH (Pv-pLDH) [6]. The SD FK80 P.f/P.v Malaria Antigen Rapid Test (Standard Diagnostics, Hagal-Dong, Republic of Korea), further referred to as FK80, is a three-band RDT that targets HRP-2 and Pv-pLDH. In areas where both *P. falciparum *and *P. vivax *are prevalent, this combination has the advantage to distinguish unequivocally between the two species, whereas a conventional three band *P. falciparum*/Pan species RDT may not. For instance, in case of a single *P. falciparum *infection, the FK80 P.f/P.v Malaria Antigen Rapid Test will only show a unique HRP-2 line; in this case the conventional three band *P. falciparum*/Pan species RDT may show (depending on the parasite density) both the HRP-2 (or Pf-pLDH line) and the pan-species line, a result which is also compatible with a *P. falciparum*/*P. vivax *mixed infection. In addition, it can be assumed that the affinity of a dedicated antibody targeting the enzyme of only a single species (Pv-pLDH in the case of *P. vivax *and the current FK80 RDT) is higher than the affinity of an antibody targeting the different enzymes of the four *Plasmodium *species (pan-pLDH). The aim of the study was to evaluate the performance of the FK80 in a non-endemic reference setting.

## Methods

### Study design

In this retrospective study, the FK80 was evaluated against a collection of stored samples obtained from international travellers, collected from January 1997 to December 2008. Tests were carried out at the malaria reference laboratory of the Institute of Tropical Medicine (ITM) Antwerp, Belgium. This study complied with the standards for the reporting of diagnostic accuracy studies (STARD) [7].

### Patients and samples

Samples were selected from a collection of EDTA-blood samples stored at -70°C and obtained from patients presenting at the outpatient clinic of ITM for clinical suspicion of malaria. The patients were international travellers and, to a lesser extent, immigrants returning from visits to their native countries. In addition, samples sent by Belgian laboratories to ITM in the scope of the national reference function were included. The samples collected at ITM, were aliquoted and frozen at -70°C the day of collection. Between collection and storage at -70°C, the samples remained at room temperature for a maximum of 10 hours, at ambient temperatures below 25°C. The 99 samples sent by Belgian laboratories to ITM for second opinion and confirmation were sent by mail and had been exposed to ambient temperature for the period of shipment, which was generally less than 24 hours and ranged to a maximum of 48 hours. The delays of shipment and processing before storage at -70°C had been validated before and were compliant with routine laboratory procedures. A representative number of *Plasmodium*-positive samples (n = 376) were selected, including the four malaria species with varying parasite densities and representative geographic distribution. Mixed infections were not included. In addition, samples without malaria parasites (as confirmed by microscopy and PCR) from symptomatic travellers suspected of malaria were included (negative samples, n = 40).

### Reference method

Microscopy corrected by polymerase chain reaction (PCR) was used as the reference method. Standard microscopy was performed on thick blood films of all samples to diagnose malaria and to assess parasite density, and on thin blood films of positive samples to define the *Plasmodium *species. Thick blood films were stained with Giemsa (pH = 8) for 20 minutes, thin films with May-Grünwald Giemsa. The slides were examined by light microscopy using a × 500 magnification, according to the standard procedure at ITM. Parasite density was assessed by counting the number of asexual parasites against 200 white blood cells in a thick film, and converting the value to parasites/μl using the actual count or the standard of 8,000 white blood cell/μl [3]. Malaria diagnosis at Central Laboratory of Clinical Biology is accredited in accordance with the requirements of the ISO 15189:2007 norm. The laboratory technicians have received a detailed training and their performance and agreement are monitored by participation to internal and external quality control assessments. As a standard procedure, all positive slides for malaria and slides showing discordant results between different laboratory tests (microscopy, RDTs or PCR) are confirmed by a blinded second microscopist. Real-time PCR analysis was adapted from Rougemont *et al *[8] as described previously [9] and performed on all samples used in this study.

### Test platform

The FK80 is lateral flow immunochromatographic RDT in a cassette format. Three lines are present, a control line which indicates whether the test is valid, a HRP-2 line and a Pv-pLDH line. According to the manufacturer's instructions, a single HRP-2 line indicates an infection with *P. falciparum*, a single Pv-pLDH line indicates an infection with *P. vivax *and a combination of a HRP-2 line and a Pv-pLDH line indicates a mixed infection with *P. falciparum *and *P. vivax*. The other *Plasmodium *species cannot be detected with this test. For the evaluation, test kits of lot number RDT 8002 (expiry date November 2010) were used.

### Test procedure

Tests were according to the instructions of the manufacturer except that samples (5 μl) where loaded with a transfer pipette (Finnpipette, Helsinki, Finland) instead of the plastic loop supplied by the manufacturer and that a scoring system was used to assess the intensity of the test lines. In case the control line did not appear, the result was interpreted as invalid and the test was repeated. In order to score test line intensities, the scoring system of Bell and co-workers was adapted [10] and five categories were defined: none (no line visible), faint (barely visible line), weak (paler than the control line), medium (equal to the control line) or strong (stronger than the control line) [9]. To assure timely readings, tests were carried out in time-controlled batches of ten samples. Readings were carried out at daylight assisted by a standard electricity bulb, between 20 and not beyond 30 minutes after application of the sample and buffer. Readings were performed by three subsequent observers, of whom the one who performed the test procedure was the first. Observers were blinded to the results of microscopy, PCR and to each others' readings. The results of the readings considered were based on consensus agreement, which means that a positive result was defined as a result read positive by at least two out of three different observers. When there was no consensus, results of the first observer were considered. Inter-reader reliabilities were assessed for the test results expressed as positive and negative readings as well as for the intensity readings. To assess test reproducibility, a panel of 19 samples (including nine *P. falciparum *samples, eight *P. vivax *samples, one *P. ovale *sample and one *P. malariae *sample) was tested on three successive occasions.

### Definitions

Samples infected with *P. falciparum *and *P. vivax *species were considered separately: Tables 1 and 2 list the definitions of true positive and negatives and those of species mismatches. In the case of *P. falciparum*, samples with pure gametocytaemia were included among the positive samples.

**Table 1:** Interpretation of test results of the FK80 for the detection of *P. falciparum*

**Test Line(s) visible**	**Species identification by microscopy corrected by PCR**	
	
	***P. falciparum***	***P. vivax, P. ovale, P. malariae*** **or no parasites detected**
		
Only HRP-2	True positive	False positive or species-mismatch*
None or only Pv-pLDH or HRP-2 + Pv-pLDH	False negative or species-mismatch**	True negative

*Non-*falciparum *species diagnosed as *P. falciparum*

**P. falciparum diagnosed as *P. vivax or as P. falciparum/P. vivax mixed infection*

**Table 2:** Interpretation of test results of the FK80 for the detection of *P. vivax*

**Test Line(s) visible**	**Species identification by microscopy corrected by PCR**	
	
	***P. vivax***	***P. falciparum, P. ovale, P. malariae *or no parasites detected**
		
Only Pv-pLDH	True positive	False positive or species-mismatch*
None or only HRP-2 or both HRP-2 and Pv-pLDH	False negative or species-mismatch**	True negative

*Non-*vivax *species diagnosed as *P. vivax*

**P. vivax diagnosed as *P. falciparum or as P. falciparum/P. vivax mixed infection*

### Statistical analysis

Sensitivity and specificity were calculated for both *P. falciparum *and *P. vivax *with 95% confidence intervals (CI) and differences were tested for significance using the Pearson chi-square test or, when this was not possible, the Fisher exact probability test. A p-value of < 0.05 was considered as significant. Reliabilities for positive and negative readings and line intensities were calculated as percentage agreements for all three readers and kappa values for each pair of readers. Associations between line intensity readings and parasite densities were assessed for strength of association with Cramer's *V *for categorical variables.

### Ease of use

Three experienced laboratory technicians scored the ease of use of the FK80 test and the clarity of manufacturer's instructions with a standardized list.

### Ethical review

The study was reviewed and approved by the Institutional Review Board of ITM and by the Ethical Committee of Antwerp University, Belgium.

## Results

### Sample collection

A total of 416 samples were selected, of which 99 samples were sent by Belgian laboratories to ITM for second opinion and confirmation. The samples were collected from January 1997 to December 2008. They were obtained in 416 patients, with a male-to-female ration of 2.19:1, and median age of 38.5 years (range 1 - 84 years). Only a minority (eight patients, 1.9%) were children less than five years old.

According to microscopy and after correction with PCR analysis, 178 of these samples were positive for *P. falciparum*, 99 for *P. vivax*, 75 for *P. ovale *and 24 for *P. malariae*. The results of microscopy were corrected in 11 out of the 376 positive samples (2.9%) and were uniquely related to *P. vivax - P. ovale *mismatches: in the final collection, four out of 99 (4.0%) *P. vivax *samples and seven out of 74 (9.5%) *P. ovale *samples had been identified as *P. ovale *and *P. vivax *by microscopy. In addition, 40 microscopic and PCR negative samples of symptomatic travellers were included in the panel.

### Invalid test results

One of the 416 samples gave an invalid result at initial testing. After 30 minutes, there was no control line visible. Upon repetition, the test performed well.

### Sensitivity and specificity

RDTs were performed between January and February 2009. Table 3 lists the test results of all samples. Test characteristics matched with species identification and parasite density for the detection of *P. falciparum *and *P. vivax *are listed in tables 4 and 5 respectively. For the detection of *P. falciparum *the sensitivity was 91.6% and the specificity was 99.2% for all samples combined. Sensitivity was higher at higher parasite densities, but the differences did not reach statistical significance. For the detection of *P. vivax *the sensitivity was 75.8% and the specificity was 100% for all samples combined. Sensitivity at parasite densities ≤ 500/μl was significantly lower compared to sensitivity at parasite densities >500/μl (51.6% and 86.8% respectively (p < 0.001)). Eight out of the 416 samples resulted in a species mismatch, with 4/178 (2.2%) *P. falciparum *samples showing a Pv-pLDH line in addition to the HRP-2 line: three of them had parasite densities higher than 1% (69,953; 87,149 and 1,000,000/μl respectively) and all four patients were upon return from Africa (travel destination were Nigeria (n = 3) and Cameroon). Another four out of 199 (2.0%) non-*falciparum *samples showed a HRP-2 line (of which two *P. vivax *samples in addition to the Pv-pLDH line) (Table 3). None of the *P. ovale *samples showed positive readings with the Pv-pLDH line.

**Table 3:** Test results for the FK80 for all samples (n = 416)

Sample	HRP-2 line positive		HRP-2 line negative		Total
			
	Pv-pLDH line positive	Pv-pLDH line negative	Pv-pLDH line positive	Pv-pLDH line negative	
					
*P. falciparum*	4*	163		11	178
*P. vivax*	2*		75	22°	99
*P. ovale*		1*		74	75
*P. malariae*		1*		23	24
Negative				40	40
Total	6	165	75	170	416

*Species mismatch

**Table 4:** Test characteristics of the FK80 for the detection of *P. falciparum *related to parasite densities (n = 416)

**Species**	**Numbers**	Correctly identified by SDFK80*	**Sensitivity in % (95% CI)**	**Specificity in % (95% CI)**
				
All *P. falciparum *samples	178	163	91.6 (86.2-95.0)	
Pure gametocytaemia	22	17	77.3 (54.2-91.3)	
Parasite density 0-100/μl	26	23	88.5 (68.7-97.0)	
Parasite density 101-1,000/μl	24	22	91.7 (71.5-98.5)	
Parasite density >1,000/μl	106	101	95.3 (88.8-98.3)	
				
Parasite density >100/μl	130	123	94.6 (88.8-97.6)	
				
Non-*falciparum *or no parasites seen	238	2**		99.2 (96.7-99.9)

*Unique HRP-2 line visible

**One *P. ovale *and one *P. malariae *sample showed a unique HRP-2 line

**Table 5:** Test characteristics of the FK80 for the detection of *P. vivax *according to parasite densities (n = 416)

**Species**	**Numbers**	Correctly identified by SDFK80*	**Sensitivity in %** **(95% CI)**	**Specificity in %** **(95% CI)**
				
All *P. vivax *samples	99	75	75.8 (65.9-83.6)	
Parasite density 0-500/μl	31	16	51.6 (33.4-62.4)	
Parasite density 501-1,000/μl	9	9	100 (62.9-100)	
Parasite density >1,000/μl	59	50	84.7 (72.5-92.4)	
Parasite density >500/μl	68	59	86.8 (75.4-93.4)	
				
Non-*vivax *or no parasites seen	317	0		100 (98.5-100)

*Unique Pv-pLDH line visible

### Line intensities

For the HRP-2 and Pv-pLDH lines, respectively 81.4% (136/167) and 55.8% (43/77) of the true positive results were read as medium or strong line intensities. For HRP-2, faint lines occurred only in three samples: one *P. falciparum *sample with pure gametocytaemia and two non-*falciparum *samples. For Pv-pLDH, faint lines occurred only in eight samples: six *P. vivax *samples (four of them with a parasitemia lower than 500 parasites/μl) and two *P. falciparum *samples. Line intensity readings for both HRP-2 and Pv-pLDH lines were related to parasite densities (HRP-2: *V *= 0.366, p < 0.001; Pv-pLDH: *V *= 0.448, p < 0.001), but there was a considerable overlap.

### Inter-reader reliability

For the HRP-2 line, the inter-reader reliability for positive and negative test results was excellent, with 98.0% agreement between the three readers and kappa values between the different pairs of readers of 0.99, 0.97 and 0.99 respectively. For the Pv-pLDH line, the inter-reader reliability for positive and negative test results was also excellent, with 98.3% overall agreement and kappa values of 0.97, 0.97 and 0.97 for the different pairs of readers.

In terms of line intensity readings, the overall agreement for the HRP-2 line was 90.2%, with kappa values of 0.88, 0.91 and 0.88 for the three pairs of readers. For the Pv-pLDH intensity readings, there was a 91.6% overall agreement with kappa values of 0.80, 0.91 and 0.80 between the three pairs of readers.

### Reproducibility

With regard to line intensity readings, HRP-2 lines were consistently read upon three times repetition for 14 out of 19 samples. All non-consistent samples had identical test results upon two times repetition and for a single sample there was a difference of two categories in line intensity (weak versus negative). Pv-pLDH intensity readings were consistent upon three times repetition for 17 out of 19 samples. The two remaining samples had consistent results upon two times repetition, and one of them had a single negative result versus two strong line intensity results. In terms of test results, this meant that two *P. falciparum *samples and a single *P. vivax *sample gave false-negative results in one out of three test repetitions.

### Ease of use

The FK80 was scored as easy to use and the instructions were scored as clear and simple to perform by all three technicians. The test was evaluated as practical and the clearance of the test strip was good. However, the clearance of the test strip was incomplete after the 15 minutes minimal incubation time recommended by the manufacturer. Good clearance was observed only after 20 minutes of waiting time.

## Discussion

The present study evaluated the performance of the FK80 against a panel of whole blood samples from international travellers suspected of malaria. The retrospective design of the study has its limitations. For instance, whole blood specimens stored at -70°C were used, and the stability of the target antigens under freezing conditions has been questioned [11]. However, no obvious differences in test performance were presently found for samples stored for several (> 5) years compared to those stored for a shorter periods (results not shown). In addition, a prospective evaluation of fresh and stored samples revealed similar results in case of the HRP-2 antigen detection [12]. Another limitation is the fact that a calibrated pipette was used for the transfer of the blood; with an expected better accuracy as compared to the kit's application loop. In addition, the diagnosis and evaluation were carried out in a reference setting, which makes extrapolation of the present results to field settings difficult [4,11]. Likewise, the ease of use was checked by an expert team and not by untrained end users in remote areas, and it is known that expert technicians tend to score tests kits more favorably [13].

The performance of the FK80 can best be compared to other tests by considering the HRP-2 and Pv-pLDH lines separately. For the HRP-2 test line in the diagnosis of *P. falciparum*, the sensitivities were in line with those reported in other HRP-2 tests in returned travellers, with sensitivities ranging from 80% tot 99%, depending on the setting and parasite densities [2,14-20]. However, the FK80 did not meet the 95% sensitivity at 100 parasites/μl as recommended by the WHO [21]. Concerning the diagnosis of *P. vivax*, comparison is more difficult. There are three reports on the performance of the Pv-pLDH detection system in the SD FK70 test, a two-band *P. vivax *RDT marketed by the same company. Two field studies reported sensitivities of 96.4% and 93.4% respectively, with lower values at low parasite densities [22,23]. In addition, the FK70 was evaluated in the present reference setting on stored whole blood samples from travellers [6]: this study demonstrated an overall sensitivity of 88.0%, with sensitivities at parasite densities below and above 500/μl of 64.3% and 97.2% respectively. Comparison of the sensitivities of the FK80 Pv-pLDH detection with those reported for the pan-pLDH target are even more difficult, as the latter range from 1.5% to 97.0% [24,25]. Compiled sensitivities for the BinaxNOW^® ^kit for the diagnosis of *P. vivax *was reported as 69.6% [25]. With regard to specificity, there was no reaction of any of the *P. ovale *samples with the Pv-pLDH test line, but two *P. vivax *samples gave positive readings with the HRP-2 line in addition to the Pv-pLDH line. Of note is the Pv-pLDH positive result in four *P. falciparum *samples: in all cases PCR as well as travel destination argued against *P. vivax *co-infection, and three of them occurred at higher parasite densities. This is in line with earlier observations, in which occasional cross-reaction of *P. falciparum *at high parasite densities was observed in the FK70 [6].

The HRP-2 lines generally were of higher intensities as compared to the Pv-pLDH lines. The higher line intensities of the HRP-2 line as compared to the pan-pLDH and aldolase lines have been described previously [9,26]. For both the test lines, the line intensities of true positive results were higher as compared to the previously evaluated FK60 and FK70 tests [6,9].

The intended area of use of the presently evaluated FK80 RDT is confined to regions with both *P. falciparum *and *P. vivax *infections, such as Afghanistan or Korea. In such situation, the FK80 has an advantage over the traditional three-band combination RDTs that detect HRP-2 and pan-species specific pLDH of aldolase: unlike the FK80, their design does not allow to exclude a mixed infection in the case of *P. falciparum *samples that react with both the HRP-2 and the pan-species lines. The FK80 by virtue of its specific Pv-pLDH is able to distinguish accurately between infections caused by *P. falciparum*, *P. vivax *and both species combined. Accurate diagnosis of *P. vivax *is important in view of the need for specific therapy but may also be interesting because of the study of *P. vivax *malaria, because this infection may be serious [27], requires a specific treatment and is also notorious because of its high transmissibility [28,29]. In other regions where these two species co-exist, the presence of *P. ovale *and *P. malariae *can not be excluded and a three-band test with a pan-species pLDH will be a better choice. In the setting of travel medicine, the FK80 may help in distinguishing mixed *P. falciparum *- *P. vivax *infections and in the distinction between *P. vivax *and *P. ovale*. However, as in this setting cost is not a prohibitive factor, a standard three-band RDT followed by, in case of single pan-pLDH positivity, a two-band Pv-pLDH RDT such as the FK70 is an alternative to support the microscopic diagnosis between *P. ovale *and *P. vivax *[6].

In conclusion, the FK80 performed satisfactorily for the diagnosis of *P. falciparum *and *P. vivax *infections in a non-endemic setting, especially at higher parasite densities.

## List of abbreviations

Ag: Antigen; CI: Confidence interval; DNA: Desoxy-ribonucleic acid; EDTA: Ethylene diamine tetra-acetic Acid; FHML: Faculty of Health Medicine and Life Sciences, Maastricht, The Netherlands; FK60: SD FK60 Malaria Ag *P. falciparum*/Pan test (Standard Diagnostics), a three-band "one-step" malaria rapid diagnostic test for the detection of *P. falciparum *and non-*falciparum *species, targeting the HRP-2 and pLDH antigens; FK70: SD FK70 Malaria Ag. *P. vivax *test (Standard Diagnostics), a two-band "one-step" malaria rapid diagnostic test for the detection of *P. vivax*, targeting the Pv-pLDH antigen; FK80: SD FK80 Malaria Ag. *P. falciparum/P. vivax *test (Standard Diagnostics), a three-band "one step" malaria rapid diagnostic test for the detection of *P. falciparum *and *P. vivax*, targeting the HRP-2 and Pv-pLDH antigens; HRP-2: Histidine rich protein-2; ITM: Institute of Tropical Medicine, Antwerp, Belgium; RDT(s): Rapid diagnostic test(s); P.: *Plasmodium; *Pan-pLDH: Pan-specific parasite lactate dehydrogenase; PCR: Polymerase chain reaction; Pf-pLDH: *Plasmodium falciparum*-specific parasite lactate dehydrogenase; pLDH: Parasite lactate dehydrogenase; Pv-pLDH: *Plasmodium vivax*-specific parasite lactate dehydrogenase; STARD: Standards for the reporting of diagnostic accuracy studies.

## Competing interests

The authors declare that they have no competing interests.

## Authors' contributions

PG, DvD and JJ designed the study protocol. MvE and EB organized prospective sample collection. DvD and PG carried out the test evaluations, LC performed PCR analysis. PG, DvD and JJ analysed and interpreted the results and drafted the manuscript, PG and DvD performed statistical analysis. All authors contributed to the discussion of the results and the redaction of the manuscript, they all approved the final manuscript.

## References

[B1] Hanscheid T Current strategies to avoid misdiagnosis of malaria Clin Microbiol Infect 2003 9 497 504 10.1046/j.1469-0691.2003.00640.x 12848724

[B2] Marx A Pewsner D Egger M Nuesch R Bucher HC Genton B Hatz C Juni P Meta-analysis: accuracy of rapid tests for malaria in travelers returning from endemic areas Ann Intern Med 2005 142 836 846 15897534 10.7326/0003-4819-142-10-200505170-00009

[B3] Moody A Rapid diagnostic tests for malaria parasites Clin Microbiol Rev 2002 15 66 78 10.1128/CMR.15.1.66-78.2002 11781267 PMC118060

[B4] Murray CK Gasser RA Jr Magill AJ Miller RS Update on Rapid Diagnostic Testing for Malaria Clin Microbiol Rev 2008 21 97 110 10.1128/CMR.00035-07 18202438 PMC2223842

[B5] Wongsrichanalai C Barcus MJ Muth S Sutamihardja A Wernsdorfer WH A review of malaria diagnostic tools: microscopy and rapid diagnostic test (RDT) Am J Trop Med Hyg 2007 77 119 127 18165483

[B6] Gillet P Bosselaers K Cnops L Bottieau E Van Esbroeck M Jacobs J Evaluation of the SD FK70 malaria Ag *Plasmodium vivax *rapid diagnostic test in a non-endemic setting Malar J 2009 8 129 10.1186/1475-2875-8-129 19519915 PMC2700805

[B7] Bossuyt PM Reitsma JB Bruns DE Gatsonis CA Glasziou PP Irwig LM Moher D Rennie D de Vet HC Lijmer JG The STARD statement for reporting studies of diagnostic accuracy: explanation and elaboration. The Standards for Reporting of Diagnostic Accuracy Group Croat Med J 2003 44 639 650 14515429

[B8] Rougemont M Van Saanen M Sahli R Hinrikson HP Bille J Jaton K Detection of four Plasmodium species in blood from humans by 18S rRNA gene subunit-based and species-specific real-time PCR assays J Clin Microbiol 2004 42 5636 5643 10.1128/JCM.42.12.5636-5643.2004 15583293 PMC535226

[B9] Palen M Van der Gillet P Bottieau E Cnops L Van Esbroeck M Jacobs J Test characteristics of two rapid antigen detection tests (SD FK50 and SD FK60) for the diagnosis of malaria in returned travellers Malar J 2009 8 90 10.1186/1475-2875-8-90 19416497 PMC2688521

[B10] Bell DR Wilson DW Martin LB False-positive results of a *Plasmodium falciparum *histidine-rich protein 2-detecting malaria rapid diagnostic test due to high sensitivity in a community with fluctuating low parasite density Am J Trop Med Hyg 2005 73 199 203 16014858

[B11] Bell D Peeling RW Evaluation of rapid diagnostic tests: malaria Nat Rev Microbiol 2006 4 S34 S38 10.1038/nrmicro1524 17034070

[B12] Mayxay M Pukrittayakamee S Chotivanich K Looareesuwan S White NJ Persistence of *Plasmodium falciparum *HRP-2 in successfully treated acute falciparum malaria Trans R Soc Trop Med Hyg 2001 95 179 182 10.1016/S0035-9203(01)90156-7 11355555

[B13] Seidahmed OM Mohamedein MM Elsir AA Ali FT Malik eF Ahmed ES End-user errors in applying two malaria rapid diagnostic tests in a remote area of Sudan Trop Med Int Health 2008 13 406 409 18298604 10.1111/j.1365-3156.2008.02015.x

[B14] De Monbrison F Gerome P Chaulet JF Wallon M Picot S Peyron F Comparative diagnostic performance of two commercial rapid tests for malaria in a non-endemic area Eur J Clin Microbiol Infect Dis 2004 23 784 786 10.1007/s10096-004-1202-9 15452770

[B15] Durand F Crassous B Fricker-Hidalgo H Carpentier F Brion JP Grillot R Pelloux H Performance of the Now Malaria rapid diagnostic test with returned travellers: a 2-year retrospective study in a French teaching hospital Clin Microbiol Infect 2005 11 903 907 10.1111/j.1469-0691.2005.01253.x 16216106

[B16] Farcas GA Zhong KJ Lovegrove FE Graham CM Kain KC Evaluation of the Binax NOW^® ^ICT test versus polymerase chain reaction and microscopy for the detection of malaria in returned travelers Am J Trop Med Hyg 2003 69 589 592 14740873

[B17] Gatti S Gramegna M Bisoffi Z Raglio A Gulletta M Klersy C Bruno A Maserati R Madama S Scaglia M A comparison of three diagnostic techniques for malaria: a rapid diagnostic test (NOW Malaria), PCR and microscopy Ann Trop Med Parasitol 2007 101 195 204 10.1179/136485907X156997 17362594

[B18] Grobusch MP Hanscheid T Gobels K Slevogt H Zoller T Rogler G Teichmann D Comparison of three antigen detection tests for diagnosis and follow-up of falciparum malaria in travellers returning to Berlin, Germany Parasitol Res 2003 89 354 357 12632146 10.1007/s00436-002-0764-7

[B19] Jelinek T Grobusch MP Schwenke S Steidl S von Sonnenburg F Nothdurft HD Klein E Loscher T Sensitivity and specificity of dipstick tests for rapid diagnosis of malaria in nonimmune travelers J Clin Microbiol 1999 37 721 723 9986839 10.1128/jcm.37.3.721-723.1999 PMC84534

[B20] Ende J Van Den Vervoort T Van Gompel A Lynen L Evaluation of two tests based on the detection of histidine rich protein 2 for the diagnosis of imported Plasmodium falciparum malaria Trans R Soc Trop Med Hyg 1998 92 285 288 10.1016/S0035-9203(98)91013-6 9861398

[B21] World Health Organization Regional Office for the Western Pacific 2003. Malaria Rapid Diagnosis: Making it Work Meeting report 20-23 January 2003. Manila, the Philippines 2003 http://www.searo.who.int/LinkFiles/Malaria_MalariaRDT.pdf

[B22] Kim SH Nam MH Roh KH Park HC Nam DH Park GH Han ET Klein TA Lim CS Evaluation of a rapid diagnostic test specific for *Plasmodium vivax* Trop Med Int Health 2008 13 1495 1500 10.1111/j.1365-3156.2008.02163.x 18983278

[B23] Lee SW Jeon K Jeon BR Park I Rapid diagnosis of vivax malaria by the SD Bioline Malaria Antigen test when thrombocytopenia is present J Clin Microbiol 2008 46 939 942 10.1128/JCM.02110-07 18160449 PMC2268337

[B24] Coleman RE Maneechai N Rachapaew N Kumpitak C Soyseng V Miller RS Thimasarn K Sattabongkot J Field evaluation of the ICT Malaria Pf/Pv immunochromatographic test for the detection of asymptomatic malaria in a *Plasmodium falciparum/vivax *endemic area in Thailand Am J Trop Med Hyg 2002 66 379 383 12164291 10.4269/ajtmh.2002.66.379

[B25] Murray CK Bell D Gasser RA Wongsrichanalai C Rapid diagnostic testing for malaria Trop Med Int Health 2003 8 876 883 10.1046/j.1365-3156.2003.01115.x 14516298

[B26] Richter J Gobels K Muller-Stover I Hoppenheit B Haussinger D Co-reactivity of plasmodial histidine-rich protein 2 and aldolase on a combined immuno-chromographic-malaria dipstick (ICT) as a potential semi-quantitative marker of high *Plasmodium falciparum *parasitaemia Parasitol Res 2004 94 384 385 10.1007/s00436-004-1213-6 15549388

[B27] Barnadas C Ratsimbasoa A Tichit M Bouchier C Jahevitra M Picot S Menard D *Plasmodium vivax *resistance to chloroquine in Madagascar: clinical efficacy and polymorphisms in pvmdr1 and pvcrt-o genes Antimicrob Agents Chemother 2008 52 4233 4240 10.1128/AAC.00578-08 18809933 PMC2592859

[B28] Phimpraphi W Paul RE Yimsamran S Puangsa-art S Thanyavanich N Maneeboonyang W Prommongkol S Sornklom S Chaimungkun W Chavez IF Blanc H Looareesuwan S Sakuntabhai A Singhasivanon P Longitudinal study of *Plasmodium vivax *and *Plasmodium falciparum *in a Karen population in Thailand Malar J 2008 7 99 10.1186/1475-2875-7-99 18518964 PMC2443811

[B29] Teka H Petros B Yamuah L Tesfaye G Elhassan I Muchohi S Kokwaro G Aseffa A Engers H Chloroquine-resistant *Plasmodium vivax *malaria in Debre Zeit, Ethiopia Malar J 2008 7 220 10.1186/1475-2875-7-220 18959774 PMC2584068

